# Exploring the Spatial Arrangement of Simple 18-Membered Hexaazatetraamine Macrocyclic Ligands in Their Metal Complexes

**DOI:** 10.3390/ijms25126802

**Published:** 2024-06-20

**Authors:** Julio Corredoira-Vázquez, Cristina González-Barreira, Jesús Sanmartín-Matalobos, Ana M. García-Deibe, Matilde Fondo

**Affiliations:** 1 Departamento de Química Inorgánica, Facultade de Química, Campus Vida, Universidade de Santiago de Compostela, 15782 Santiago de Compostela, Spain; julio.corredoira.vazquez@usc.es (J.C.-V.); cristina.gonzalez.barreira@rai.usc.es (C.G.-B.); jesus.sanmartin@usc.es (J.S.-M.); 2 Phantom-g, CICECO—Aveiro Institute of Materials, Department of Physics, University of Aveiro, 3810-193 Aveiro, Portugal; 3 Institute of Materials (iMATUS), Universidade de Santiago de Compostela, 15782 Santiago de Compostela, Spain

**Keywords:** *N*_6_ macrocycle, amine ligands, conformation, twisting, dysprosium, pyridine, fluorescence

## Abstract

Hexaazamacrocyclic Schiff bases have been extensively combined with lanthanoid (Ln) ions to obtain complexes with a highly axial geometry. However, the use of flexible hexaazatetraamine macrocycles containing two pyridines and acyclic spacers is rather uncommon. Accordingly, we obtained [DyL(OAc)_2_]OAc·7H_2_O·EtOH and [DyL^Me2^(Cl)_2_]Cl·2H_2_O, where L and L^Me2^ are the 18-membered macrocycles 3,6,10,13-tetraaza-1,8(2,6)-dipyridinacyclotetradecaphane and 3,10-dimethyl-3,6,10,13-tetraaza-1,8(2,6)-dipyridinacyclotetradecaphane, respectively, which contain ethylene and methylethylene spacers between their *N*_3_ moieties. [DyL(OAc)_2_]OAc·7H_2_O·EtOH represents the first crystallographically characterized lanthanoid complex of L, while [DyL^Me2^(Cl)_2_]Cl·2H_2_O contributes to increasing the scarce number of Ln^III^ compounds containing L^Me2^. Furthermore, the crystal structure of L·12H_2_O was solved, and it was compared with those of other related macrocycles previously published. Likewise, the crystal structures of the Dy^III^ complexes were compared with those of the lanthanoid and *d*-metal complexes of other 18-membered *N*_6_ donor macrocycles. This comparison showed some effect of the spacers employed, as well as the influence of the size of the ancillary ligands and the metal ion. Additionally, the distinct folding behaviors of these macrocycles influenced their coordination geometries. Moreover, the luminescent properties of [DyL(OAc)_2_]OAc·7H_2_O·EtOH and [DyL^Me2^(Cl)_2_]Cl·2H_2_O were also investigated, showing that both complexes are fluorescent, with the emission being sensitized by the ligands.

## 1. Introduction

Hexaazamacrocycles are Lewis bases that are widely used in coordination chemistry [1]. Among them, those holding pyridine rings are especially abundant [2]. Regarding their coordination with lanthanoids (Ln), although Ln^III^ ions are considered hard Pearson acids with a preference for ligands with hard donor atoms as oxygen, these types of multidentate N donors have been broadly employed to obtain numerous complexes [3]. Thus, the macrocyclic effect makes lanthanoids highly prone to be coordinated with these ligands, which can also hold varied podands [2]. In the absence of these complexing arms, the number and features of ancillary ligands significantly influence the total coordination number of the central metal atom. Thus, it has been observed that when ancillary ligands are small, the coordination number commonly exceeds 8, while with bulky ancillary ligands, a coordination number of 8 is much more common [3].

**Scheme 1:**
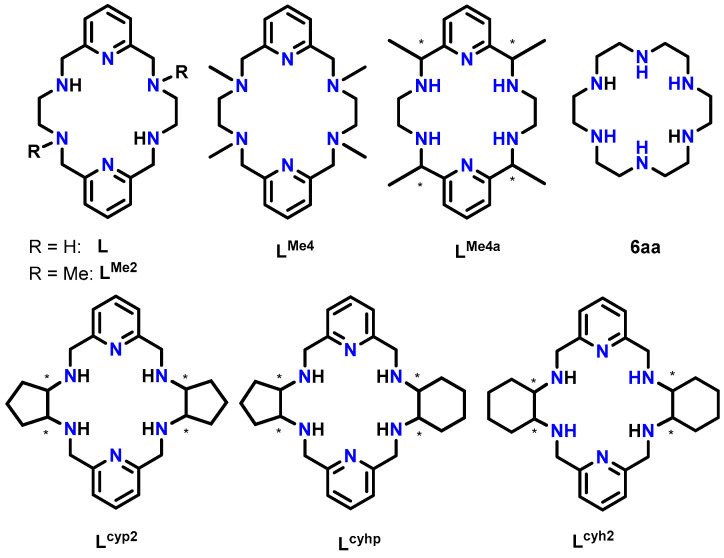
The 18-membered hexaazamacrocyclic ligands used in this work (L and L^Me2^) as well as some other related ligands mentioned. All of them (L^x^) are depicted as neutral species, and obviating any chirality of the asymmetric C atoms is marked with *.

The coordination geometry is always a remarkable aspect, especially for properties such as magnetism and luminescence. Its dependence on the flexibility of the macrocycle is evident, although the influence of this parameter on the geometry adopted by lanthanoid ions in their complexes has not been extensively investigated. To the best of our knowledge, a considerable number of Ln compounds with rigid or semi-rigid polyimine *N*_6_ macrocycles derived from 2,6-pyridinedicarboxaldehide have been crystallographically characterized [3]. In contrast, the use of flexible polyamines including two rigid pyridine residues, with different aliphatic spacers and lacking long podands, is rather uncommon [3]. In fact, if we limit our focus to 18-membered macrocycles of this latter type, such as the tetraamine macrocycles shown in Scheme 1, only a very limited number of metal complexes have been crystallographically reported [4,5,6,7,8,9,10,11,12,13,14,15].

A search in the Cambridge Structural Database (CSD) [16] (5.45 version of November 2023, update of March 2024) revealed that multiple crystal structures are known for the free hexaazatetraamine ligands represented in Scheme 1, which we can name L^x^, regardless of their degree of protonation, so they include amine and/or ammonium species. In contrast, only metal complexes are deposited in this database for L, L^Me2^, L^Me4^ L^Me4a^, and L^cyh2^. Thus, some divalent *d*-metal (Co, Ni, Cu, Zn, and Cd) complexes of the L, L^Me4^, L^Me4a^, and L^cyh2^ macrocycles have been crystallographically characterized [10,11,12,13,14,15]. These complexes lack additional ligands, with their *d*-block metal ions showing distorted octahedral geometries formed by its six *N* donor atoms.

In addition, some lanthanoid complexes with this kind of 18-membered *N*_6_ macrocycles were also reported. Among them, most of the crystal structures solved contain L^cyh2^ (Scheme 1), which displays cyclohexyl spacers between the two *N*_3_ donor groups. This ligand has been found to coordinate with La, Pr, Sm, Eu, Tb, Dy, Ho, Er, Tm, Yb, and Lu, as well as Y [7,9]. However, complexes of Ln with ligands from Scheme 1 with acyclic aliphatic spacers are much less common. In fact, no crystallographically characterized derivatives are known for L and L^Me4^, while only two Ln^III^ complexes have been reported for L^Me2^ [4] and one for L^Me4a^ [8]. For these two latter ligands, the complexes contain quite large lanthanoids (Ce, Nd, or Sm), but no coordination compounds are known with smaller Ln^III^ ions. It is noteworthy that in these latter three compounds, the ligands provide a reasonably flat *N*_6_ environment for the Ln^III^ ion.

We have been recently interested in the coordination of lanthanoid ions to related semi-rigid *N*_6_ Schiff base macrocycles, with very interesting results [17]. Now, with the aim of assessing the influence of acyclic aliphatic spacers and the size of the metal ion on the geometry of the complexes, in this work, we prepared two novel dysprosium complexes, [DyL(OAc)_2_]OAc·7H_2_O·EtOH and [DyL^Me2^(Cl)_2_]Cl·2H_2_O, with two very different spatial arrangements.

As the conformation of the free ligands and their corresponding complexes is strongly influenced both by isomerism and chirality, these aspects were also taken into consideration, by comparing the crystal structure of L·12H_2_O with other related 18-membered hexaazamacrocycles collected in Scheme 1.

## 2. Results and Discussion

### 2.1. Synthesis

The L and L^Me2^ ligands were obtained by previously described procedures [18,19]. The [DyL(OAc)_2_]OAc·7H_2_O·EtOH and [DyL^Me2^(Cl)_2_]Cl·2H_2_O complexes were isolated from typical synthetic methods by mixing the free ligand and the corresponding hydrated dysprosium salt (acetate or chloride) in a 1:1 molar ratio, using MeCN and MeOH as solvents.

### 2.2. X-ray Diffraction Studies

Single crystals of L·12H_2_O, [DyL(OAc)_2_]OAc·7H_2_O·EtOH, and [DyL^Me2^(Cl)_2_]Cl·2H_2_O suitable for X-ray diffraction studies were isolated as described in the experimental section. Some aspects of the data acquisition and the resolution are recorded in Table S1.

#### 2.2.1. Ligands

**Figure 1:**
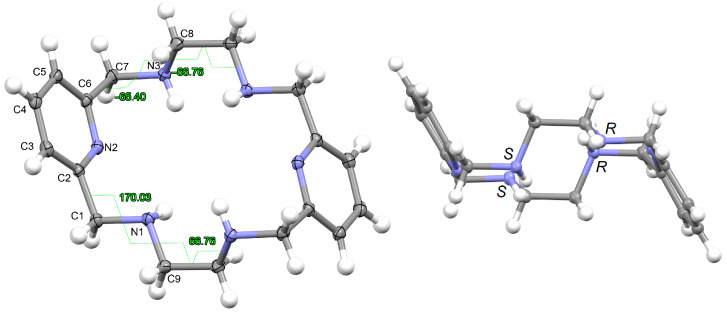
Ellipsoid diagrams (50% probability) with the labeling scheme of L in L·12H_2_O, only for atoms of the asymmetric unit and showing the opposite torsions of both ethylene spacers (**left**), as well as those of each branch (C_Py_-CH_2_-NH-CH_2_) of the acetyl pyridine residue. In the **right panel**, another view is presented of the chirality of the amine *N* donor atoms shown in the crystal, with similar chirality for the two N atoms of each diacetylpyridine residue, an opposite for each ethylenediamine one.

L·12H_2_O crystallizes as a neutral species in the triclinic space group *P*-1. Its asymmetric unit only contains half a molecule of the ligand, along with six water molecules as solvates, being the whole molecule generated by an inversion center (1 − *x*, 2 − *y*, *z*). The neutral tetraamine displays two of the four amine H atoms in equatorial positions (N3) and the other two ones with an axial disposition (N1). The solvated water molecules are all interconnected by an intricate H-bonding system, which involves the amine groups. Ellipsoid diagrams with two different orientations are shown in Figure 1 to illustrate the spatial arrangement of the macrocycle.

To facilitate the comparison of this crystal structure, Table 1 collects some aspects related to all the reported crystal structures of different species of the free ligands shown in Scheme 1, such as symmetry, isomerism, or chirality. CSD_REFCOD is also included to identify the crystal data deposited in the Cambridge Structural Data.

The molecule displays bond distances and angles (Table S2) in the ranges expected, if compared with some related species depicted in Scheme 1, as well as with neutral macrocycles L^Me4a^·2H_2_O (CSD_KUBCOA) [12,20] and L^cyh2^·2CHCl_3_ (CSD_RIVPIX) [7]. These parameters are also comparable to those of two different tetraammonium (H_4_L)^4+^ species, namely [H_4_L](NO_3_)_4_·(CSD_ZUVKUW) [21] and [H_4_L]Br_4_·H_2_O (CSD_ZIVPAV) [22]. They are also in the range of those found for other tetraprotonated related ligands such as [H_4_L^Me2^](NO_3_)_4_ (CSD_DIJZOO) [4], [H_4_L^Me4a^]Br_4_·2H_2_O (CSD_NOQFEE) [23], [H_4_L^Me4a^](NO_3_)_4_·5H_2_O (CSD_SICCIR) [8], [H_4_L^Cyh2^]Cl_4_·6H_2_O (CSD_VISWOM) [24], [H_4_L^Cyhp^]Cl_4_·1.7MeCN·1.2MeOH·0.4H_2_O (CSD_ZOKNUL) [25], [H_4_L^N6Cyp^]Cl_4_·2MeOH (CSD_EQUGII) [26], and [H_4_L^cyp2^]Cl_4_·6.2H_2_O (CSD_HUVYOP) [27]. Likewise, diprotonated species such as [H_2_L^cyp2^]Cl_2_ (CSD_EDEWIW) [28], or [H_2_L^Me4a^](NO_3_)_2_ (CSD_SICCIR) [8] also show comparable geometric data.

**Table 1:** Conformations and chirality found for different species related to the ligands shown in Scheme 1.

Figures for the Ligand	Ligand, Counterion	CSD_REFCOD	Symmetry, Folding	C_Py_-CH_(2)_-NH_(2)_-CH_(2)_ Conformations Chirality in Each Spacer	Ref.
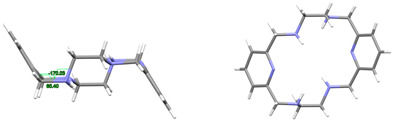	L	This work	*C_i_* Wave	Gauche and alternate (see Figure 1 and Figure S1)	This work
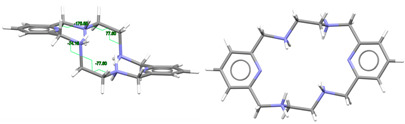	[H_4_L]^4+^, 4(Br^−^)	ZIVPAV	*C_i_* Wave	Gauche and alternate	[22]
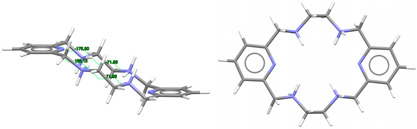	[H_4_L]^4+^, 4(NO_3_^−^)	ZUVKUW	*C_s_* Wave	Alternate and alternate	[21]
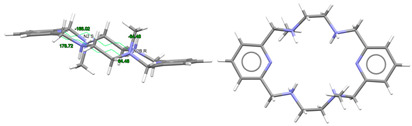	[H_4_L^Me2^]^4+^, 4(NO_3_^−^)	DIJZOO	*C_i_* Wave	Alternate and alternate *trans*, *anti* form N: *S* and N: *R*	[4]
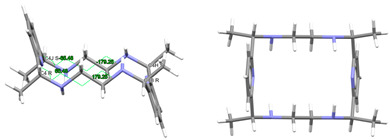	L*^meso^*^-Me4a^	KUBCOA	*C_s_* Wave	Alternate and alternate *meso*, *anti* form C: *R*,*R* and C: *S*,*S*	[12,20]
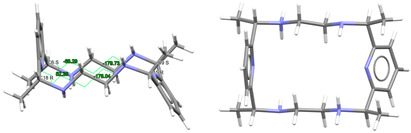	(H_2_L*^meso^*^-Me4a^)^2+^, 2(NO_3_^−^)	SICCIR	*C_s_* Wave	Alternate and alternate *meso*, *anti* form C: *R*,*R* and C: *S*,*S*	[8]
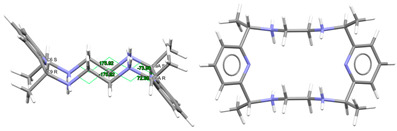	(H_4_L*^meso^*^-Me4a^)^4+^, 4(Br^−^)	NOQFEE	*C_s_* Wave	Alternate and alternate *meso*, *anti* form C: *R*,*R* and C: *S*,*S*	[23]
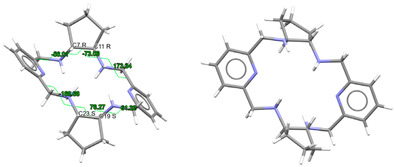	(H_2_L*^rac-^*^cyp2^)^2+^, 2(Cl^−^)	EDEWIW	*C_i_* Wave	Gauche and alternate *rac* form C: *R*,*R* and C: *S*,*S*	[28]
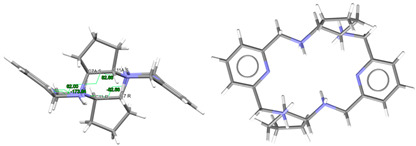	(H_4_L*^rac-^*^cyp2^)^4+^, 4(Cl^−^)	EQUGII	*C_i_* Wave	Gauche and alternate *rac* form: C: *R*,*R* and C: *S*,*S*	[26]
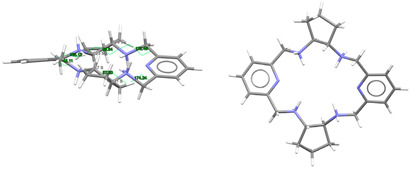	(H_4_L*^SSSS-^*^cyp2^)^4+^, 4(Cl^−^)	HUVYOP	*C*_2_ Ruffle	Alternate and alternate; *S*,*S*,*S*,*S* form C: *S*,*S* and C: *S*,*S*	[27]
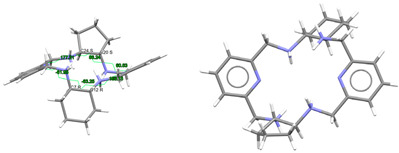	(H_4_L*^rac-^*^cyph^)^4+^, 4(Cl^−^)	ZOKNUL	*C*_1_ Wave	Gauche and alternate *rac* form C: *R*,*R* and C: *S*,*S*	[25]
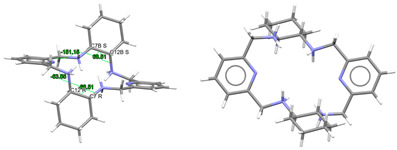	(H_4_L*^rac-^*^cyh2^)^4+^, 4(Cl^−^)	VISWOM	*C_i_* Wave	Gauche and alternate *rac* form C: *R*,*R* and C: *S*,*S*	[24]
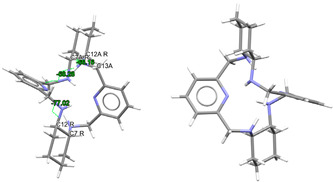	L*^RRRR^*^cyh2^	RIVPIX	*C*_2_ Ruffle	Gauche λ and λ *R*,*R*,*R*,*R* form C: *R*,*R* and C: *R*,*R*	[7]

The most significant differences are related to the molecular conformation of these free ligands, as Table 1 illustrates. As mentioned above, we were particularly interested in this kind of ligand because of its potential planar disposition around metal ions to occupy the base of a hypothetical bipyramid. In fact, hexaaza[18]annulene (6aa, Scheme 1), which is supposed to be more flexible than L^x^ ligands, shows this disposition around lanthanoid ions in, to the best of our knowledge, the two only lanthanoid complexes of this ligand, namely [Nd(6aa)(NO_3_)_3_)] [29,30] and [(6aa)Er(µ-OH)_2_Er(6aa)]^4+^ [31], which are crystallographically characterized and deposited in the CSD. This finding is in marked contrast to the more than 150 crystal structures found in the CSD [16] for lanthanoid complexes containing the equivalent *O*_6_ donor 18-crown-6 ether.

In our case, in terms of planarity, despite the rigidity provided by the two pyridine moieties, the macrocycle in L·12H_2_O is considerably folded (Figure 1), although the *N*_6_ donor set remains quite planar, with the nitrogen atoms deviating only between 0.16 and 0.22 Å from the mean calculated *N*_6_ plane. This spatial arrangement is in contrast to the theoretical and very planar *D*_3*d*_ configuration described for 18-annulene derivatives as 6aa or 18-crown-6 since it actually shows a *C_i_* symmetry, which is also typical of both 18-membered macrocycles [32]. In agreement with experimental data, Monte Carlo and molecular dynamics simulations have demonstrated that 18-crown-6 prefers the prolate *C_i_* conformation in nonpolar solvents, while in polar solvents the *D*_3*d*_ symmetry is the preferred configuration by 16-crown-6 [32]. In the present case, however, this preference was not observed since L·12H_2_O was collected in such a polar solvent as water.

With this *C_i_* symmetry, the apparent irregularity is mainly related to the opposite conformation of the two spacers connecting both pyridine rings, which are positioned outside this plane and form angles of ca. 75° with the calculated *N*_6_ plane. These two spacers display opposite torsions for the ethylene groups of the two diamine moieties, with δ and λ gauche conformations (Figure 1). Another feature related to this *C_i_* symmetry is the different conformations exhibited by the two spacers attached to each pyridine ring, which are gauche and alternate in all these cases (Table 1).

Thus, the spatial arrangement of L in L·12H_2_O is like that of [H_4_L]^4+^ in CSD_ZIVPAV [22], whose pyridine rings are not so folded, but it contrasts with [H_4_L]^4+^ in ZUVKUW [21], with *C_s_* symmetry. This difference may be related to their counterions, namely bromide and nitrate, respectively, as in the case of the [H_4_L^Me2^]^4+^ cation. Here, NO_3_^−^ acts as a counterion in CSD_DIJZOO [4], and the conformation intensely reminds of that of CSD_ZUVKUW (Table 1) but with a *C_i_* symmetry due to the two *trans* methyl substituents [21]. These substituents are also crucial for the conformation of the L^Me4a^ species. Thus, both CSD_KUBCOA [12,20], CSD_SICCIR [8], and CSD_NOQFEE [23] show the ligand in a *meso* form, with *C_s_* symmetry, regardless of the degree of protonation (0, 2, or 4).

This difference in the conformation is related to the conformation of the C_Py_-CH_(2)_-NH_(2)_-CH_(2)_ groups (Figure S1), as they differ for [H_4_L]Br_4_·H_2_O (gauche and alternate) (CSD_ZIVPAV) [21], while both are alternate for CSD_ZUVKUW [21]. Except for the dimethyl substituted CSD_DIJZOO [4] and CSD_ZOKNUL containing the asymmetric [H_4_L^cyph^]^4+^ (*C_i_*), all the L^x^ species with *C_i_* symmetry are related to simultaneous gauche and alternate conformations for the two C_Py_-CH_(2)_-NH_(2)_-CH_(2)_ groups of each pyridine.

Other ligands conformed as L in L·12H_2_O are CSD_EDEWIW [28], CSD_EQUGII [26], CSD_ZIVPAV, CSD_HUVYOP [27], CSD_ZOKNUL [20], and CSD_VISWOM [24] (Table 1), with dissimilar conformations (gauche and alternate) for their C_Py_-CH_(2)_-NH_(2)_-CH_(2)_ groups. In their case, they all contain cyclopentyl and/or cyclohexyl diamine residues, instead of ethylene groups in their spacers. Furthermore, the presence of these cycles introduces a new key factor: chirality.

From the observation of Table 1, it appears that L^x^ species with racemic spacers (*R*,*R* and *S*,*S*) tend to present a *C_i_* symmetry, being conformed as L in L·12H_2_O. In contrast, the two homochiral macrocycles L^cyh2^ in CSD_RIVPIX [7] and (H_4_L^cyp2^)^4+^ in CSD_HUVYOP [27] display a *C*_2_ symmetry. However, their appearance is very different: rather planar for (*S*,*S*,*S*,*S-*H_4_L^cyp2^)^4+^ whereas very twisted in *R*,*R*,*R*,*R-*L^cyh2^. This appears to be related to the different conformation of the C_Py_-CH_2_-NH_(2)_-CH groups attached to each pyridine ring, which are doubly alternate for CSD_HUVYOP [27] while doubly gauche-conformed for CSD_RIVPIX [7].

#### 2.2.2. Metal Complexes

With the intention of gaining a better understanding of the behavior of this kind of ligand (L^x^) toward metal ions, we reviewed the CSD files [16] to find another 20 crystal structures, including yttrium and lanthanoid complexes [4,5,6,7,8,9], in addition to [DyL(OAc)_2_]OAc·7H_2_O·EtOH and [DyL^Me2^(Cl)_2_]Cl·2H_2_O, which are presented here. These results and some parameters related to their spatial arrangement are collected in Table S3. Additionally, hexacoordinate complexes of Cd^II^ and some divalent metals of the 3*d* series are also summarized in Table S4. The ionic radii of metal centers [33,34,35] were also taken into consideration in both cases.

To simplify the qualification of their spatial arrangements, and due to the presence of the two pyridine rings, connected through aliphatic cyclic spacers in many cases, we can use the typical terminology applied to such popular macrocycles as porphyrins, with terms such as “saddle”, “ruffle”, “dome” or “wave” [36] to describe the different conformations adopted by the L^x^ species in their various crystal structures. Prior to this comparison, we will comment some of features of the crystal structures presented here.

**Figure 2:**
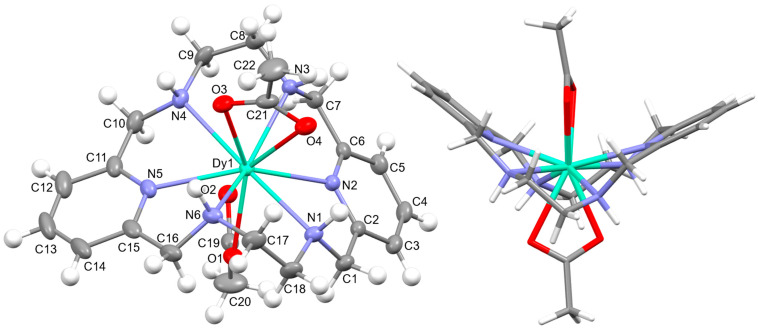
(**Left**) The ellipsoid diagram for the [DyL(OAc)_2_]^+^ cation (ellipsoids at 35% probability) showing the labeling scheme; (**Right**) the stick view from a different perspective to show the folding of the macrocycle.

[DyL(OAc)_2_]OAc·7H_2_O·EtOH: Its crystal structure reveals its ionic nature, consisting of mononuclear cations [DyL(OAc)_2_]^+^**,** plus free acetate anions and water and ethanol solvates. Ellipsoid and stick diagrams, with different perspectives, are shown in Figure 2 for the cationic complex. The main bond distances and angles around the Dy^III^ center are listed in Table S5. The Dy^III^ ion is coordinated with the six N atoms of the neutral tetraamine ligand. The coordination sphere is completed by two acetate ligands acting as bidentate chelating donors. Both anions occupy opposite sides of the macrocycle. Their respective chelate planes form an angle of ca. 72.6°. The dysprosium center of [DyL(OAc)_2_]^+^ is decacoordinate in an N_6_O_4_ environment. Calculations of the degree of distortion of the DyN_6_O_4_ core with respect to an ideal ten-vertex polyhedron made with the SHAPE 2.1 software [37] show that the best geometry corresponds to a bicapped square antiprism, distorted to a sphenocorona (Table S6).

This disposition of the ligands is similar to that found for the cations [SmL^Me2^(NO_3_)_2_]^+^ (CSD_DIJZII) [4], [CeL^Me2^(NO_3_)_2_]^+^ (CSD_DIJZEE) [4], [Dy^cyh2^(NO_3_)_2_]^+^ (CSD_YIPJEN) [9], and [YbL^cyh2^(NO_3_)_2_]^+^ (CSD_YIPJIR) [9] (Table S3). All of them contain bidentate chelating nitrate ions, which are also twisted, and where the L^x^ species are folded with both pyridine rings forming obtuse angles, while the folding occurs through their spacers. This bending causes the spacers to be on opposite sides of the bent macrocycle. Thus, they all resemble saddle-shaped conformed porphyrins [36]. In the case of [DyL(OAc)_2_]OAc·7H_2_O·EtOH, the two pyridine rings form an angle of 115.52 degrees, while this angle is close to 160° for the samarium or the cerium complexes mentioned above. In contrast, [NdL^Me4a^(NO_3_)_2_]^+^ in CSD_SICCOX [8] also shows two bidentate chelating nitrate anions but practically in parallel, while the hexaazamacrocycle displays a “waving” disposition. This conformation is apparently preserved from the free ligand [6,12,20,23] since all its crystal structures display a similar spatial arrangement (Table 1). This can be related to an evident preference of the ligand for that wave-like conformation, which is adopted by the *meso* form of L^Me4a^, in combination with the steric hindrance imposed by the four methyl groups to the ancillary anionic ligands.

L is not deprotonated after coordination, as it comprises the four amine H atoms sited at the same side of the ligand. Therefore, the four amine N atom groups are genuine stereocenters after coordination, which display the following configurations: N1: *S*, N3: *R*, N4: *S*, and N6: *R* (Figure 2). Accordingly, each spacer is racemic, being the two amine groups connected to each pyridine also heterochiral.

**Figure 3:**
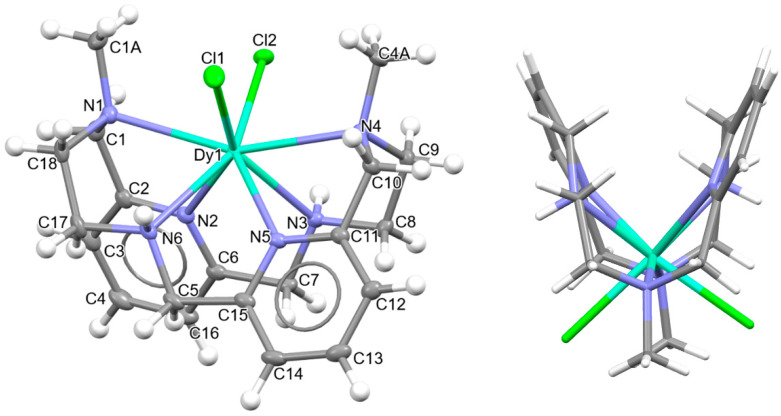
Ellipsoids and sticks diagram for the [DyL^N6Me^Cl_2_]^+^ cation. After coordination, the configurations of the chiral N atoms are N1: *R*, N3: *S*, N4: *R*, and N6: *S*.

[DyL^Me2^Cl_2_]Cl·2H_2_O: Chloride anions, [DyL^Me2Ok^Cl_2_]^+^ cations, in combination with some solvated water molecules, fill the unit cell of this crystal structure. Different types of diagrams for the cation are represented in Figure 3, while the main bond distances and angles around the Dy^III^ ion are also collected in Table S5. Regarding Dy-N, Dy-O distances as well as the angles between donor atoms about the Dy^III^ ion are in the range of the expected ones [6,7,9]. The same similarity is found for the Dy-Cl coordination [38].

In this case, the macrocycle can be considered as folded in half along the N_Me_-Dy-N_Me_ angle, leaving aside the spacers, so that the pyridine rings are not in front of each other. Thus, the chelate rings appear like the wings of a butterfly, with the methyl groups oriented toward the outside side of the macrocycle, as well as the H atoms of the other two amine groups and similarly to both [CeL^Me2^(NO_3_)_2_]^+^ in CSD_DIJZEE [4] and [SmL^Me2^(NO_3_)_2_]^+^ in CSD_DIJZII [4]. This spatial disposition can be considered a *trans*–*syn* form of the ligand, and it contrasts with the *trans–anti* species collected in Table 1 for CSD_DIJZOO [4]. Despite the coincident form of these three complexes, L^Me2^ is more open for the two decacoordinate ones with a tetradodecahedron geometry, since the dihedral angle between the pyridine units is higher than 160 in a saddle-like disposition. In contrast, the two pyridine rings of [DyL^N6Me^Cl_2_]^+^ form an angle of only ca. 45°. Such proximity between the two halves of the macrocycle allows C-H···π interactions between an H atom of each ethylene spacer and a pyridine ring (2.89 Å). This marked flexion could be related to the presence of the two contiguous chloride anions, which exhibit a *cis* configuration, in their case with an angle of about 101.6 degrees.

Despite achieving the desired octacoordination, the *cis* disposition of the two chloride anions does not lead to a hexagonal bipyramidal coordination geometry. In fact, calculations for the *DyN_6_Cl_2_* core using SHAPE [37] suggest a triangular dodecahedron as the closest regular polyhedron but with a high distortion toward a biaugmented trigonal prism (Table S6).

The observation of Table S3 shows that although [DyL^Me3^Cl_2_]^+^ is the only complex with two coordinated chloride anions, the *cis* disposition of the two monodentate ligands is not unfamiliar for this type of tetraamine ligand, as it is comparable to that shown by other related complexes with two monodentate donors as ancillary ligands. This is the case of [DyL^cyh2^(OH_2_)Cl]^2+^ in CSD_FOFBOU [6], [YbL^cyh2^(OH_2_)Cl]^2+^ in CSD_RIVQOE [7], [TmL^cyh2^(OH_2_)_2_]^3+^ in CSD_RIVQAQ [7], [LuL^cyh2^(OH_2_)_2_]^3+^ in CSD_RIVQEU [7], and even the 4*d*-metal complex [YL^cyh2^(OH_2_)Cl]^2+^ in CSD_RIVQIY [7]. The butterfly arrangement of the ligand is easier to appreciate for L^cyh2^ because of the cyclic spacer.

### 2.3. Spatial Arrangements of Metal Complexes of L^x^ Ligands

As commented above, neither [DyL(OAc)_2_]^+^ nor [DyL^Me2^Cl_2_]^+^ shows unfamiliar spatial arrangements for lanthanoid complexes with this kind of ligand. Therefore, we tried to deduce the reasons for such different behaviors for the same metal ion, as well as the possible reasons for finding only a few spatial arrangements. With this purpose, we present the results in Tables S3 and S4. Furthermore, we also tried to analyze the best fits for the cavities of these hexaazamacrocycles, both for divalent and trivalent metal cations. Therefore, we compared the structure adopted in each complex with that assumed for a regular hexagonal disposition of the six N donor atoms, as a function of their ionic radius [33,34,35], as it is known that a different degree of distortion in the macrocycles can cause affection of other properties [39]. This type of theoretical study has been satisfactorily applied to determine the relationship between the transversal magnetic anisotropy and the distortion of a hexaazamacrocycle from planarity [40] or to find the most appropriate size to fit inside the 18-crown-6 ether cavity [41]. This latter *D_3d_* planar cavity has a diameter of 5.8 Å, while for 6aa, it is 5.5 Å [32]. In this sense, L^x^ tends to be oblonger than 6aa, probably because of the presence of the rigid pyridine rings. Thus, although [H_4_L]^4+^ is rather regularly conformed in the crystal structure of CSD_ZUVKUW [20], the distances between inverted amine groups show values of 5.354(3) and 5.562(3) Å, while the two pyridine N atoms are at 6.237(5) Å. In the case of L·12H_2_O, which is more elongated, the N_py_···N_py_ distance is about 6.1 Å, while the distances between opposite amine atoms are different to such an extent as ca. 4.6 and 6.6 Å.

#### 2.3.1. Complexes of Divalent Metals of *d*-Block

**Figure 4:**
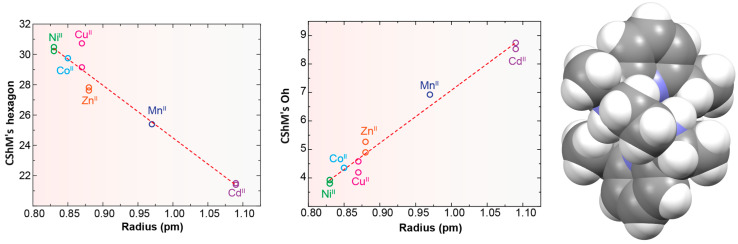
Representations of the ionic radii R (for coordination number = 6) of some divalent *d*-block metal ions vs. deviations of continuous shape measures (CShMs) from a D6h hexagon (**left**) and Oh octahedron (**center**). Spacefill representation of the *M* helical spatial arrangement of L^Me4a^ in [NiL^Me4a^]^2+^ for CSD_KUBCUG [12] (**Right**).

Probably the elongation of the cavity of L^x^, along with the good flexibility of the different spacers used, are significant factors for the adequacy of this type of ligand to form pseudo-octahedral complexes with divalent ions of the *d*-block [5,10,11,12,13,14,15], as shown in Table S4 and Figure 4. In the latter, we can see that, in general, the smaller the metal ion, the greater the distortion with respect to an ideal planar hexagon (Figure 4, left). Conversely, the smaller the ion is, the better the fit of the coordination polyhedrons to a regular octahedron (Figure 4, center). In this sense, it should be noted that some theoretical studies confirm this experimental observation, as it has even shown that Hg^II^, and even more so Pb^II^, would significantly untwist a ligand as L in their theoretical hexacoordinate complexes [13]. Therefore, the radius of the cation seems to be a key factor in this case. However, the inclusion of Cd^II^ in this group is significant, since its ionic radii (1.09 Å) are very similar to those of Ln^III^ ions, but it only achieves hexacoordination with these neutral ligands. This demonstrates once again that not only the size but also the coordination preferences are the clues to assembling a metal complex. Among those crystallographically characterized, the cation that best fits this octahedral disposition is Ni^II^.

No influence of the counterion has been reported for these complexes. Additionally, in view of the variety used, this does not appear to be a key point.

These cationic complexes are chiral, and they can be described as helicates since their amine groups are also chiral. From Table S4, we can see that all these chiral N atoms display the same configuration for each complex independently of the ligand employed and the configuration of other chiral atoms that are also present in the ligand. It should be noted that in the case of [NiL^Me4a^]^2+^ in CSD_KUBCUG [12] (Figure 4, right), the isomer of the ligand present in the complex is not the *meso* diasteroisomer (*R*,*R*;*S*,*S*) but *R*,*S*;*R*,*S*, so instead of the methyl groups being in axial positions, they are all equatorially positioned. Furthermore, a homochiral *R*,*R*,*R*,*R* N donor set is associated with a *P* helicity of the metal ion. Inversely, an *S*,*S*,*S*,*S* configuration corresponds to an *M* helical geometry (Figure 4) in all the cases collected. In particular, the four chiral C atoms of the complexes containing the L^cyh2^ ligand show an opposite configuration to that of the four chiral N atoms.

From the observation of CSD_RIVPIX [7] in Table 1, it is easy to appreciate the predisposition of this homochiral derivative to yield an octahedral geometry. In fact, it appears that the introduction of the cyclohexane residues between the amine N atoms seems to induce a less distorted octahedral geometry with respect to the ethylene spacers. The same effect seems to be caused by the introduction of methyl substituents on the amine nitrogen atoms, which disfavors the planarity of the donor set of the macrocycle.

#### 2.3.2. Complexes of Trivalent Metal Ions

In this epigraph, we include not only complexes of 4*f* metal ions but also Y^III^, which is comparable to lanthanoids in many aspects. The behavior of L^x^ toward trivalent ions is completely different from that shown toward divalent metal cations, even of comparable size, as previously mentioned. However, lanthanoids still seem to be too small for most of the hexaazamacrocycle ligands, so these ligands tend to adapt their conformation to the size of the central metal ion [9,42].

**Figure 5:**
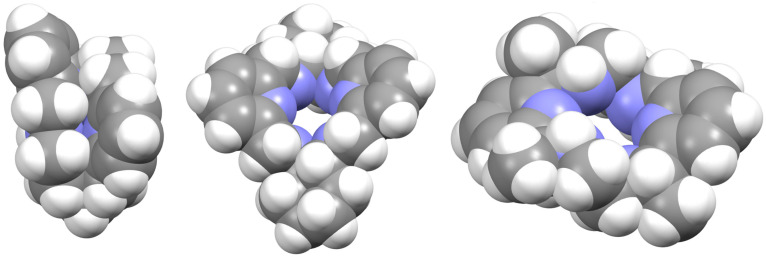
Spacefilling representations of the spatial arrangement of (from left to right): L, L^cyh2^, and L^Me4a^ in [DyL^Me2^Cl_2_]^+^, CSD_RIVRAR [7] and CSD_SICCOX [8], respectively. They try to illustrate the terms used in this work to describe the macrocycle disposition “butterfly”, “saddle” [36], and “wave” [36], respectively.

Regarding the spatial arrangements adopted by L^x^ around trivalent metal centers, and considering the content of Table S3, only three types of conformations appear among the crystallographically characterized complexes, which can be described as butterfly, saddle [36], and wave [36] (Figure 5).

**Figure 6:**
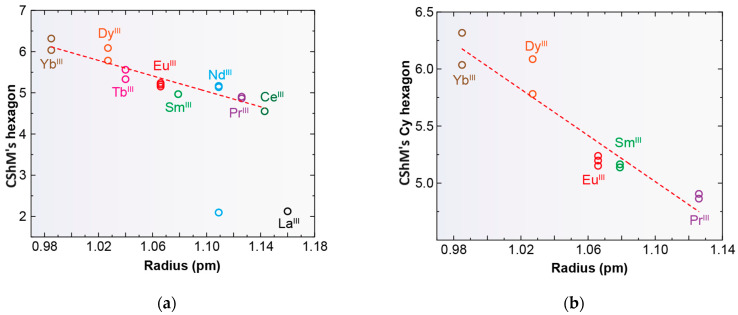
Representations of the deviations of continuous shape measures (CShMs) observed from a D_6h_ hexagon versus the ionic radii of lanthanoid metal ions for deca- and undecacoordinate complexes; (**a**) all the complexes with L^x^ and (**b**) only for complexes containing L^cyh2^ with coordination number > 9.

Only complexes with saddle or wave conformations show the *N*_6_ donor set of their macrocycles close to a planar hexagon as their best-fitting geometry (Table S3), while those with a butterfly conformation are closer to a *D_3h_* trigonal prism. Using the same calculation procedure employed above, a representation of the deviations observed for the macrocycle donor set from a regular D_6h_ hexagon in each saddle- and wave-conformed complex versus the cationic radii is shown in Figure 6. It is clear from this graph that planarity increases with increasing M^III^ radius. However, there are two clear discordances: [LaL^cyh2^(NO_3_)_3_] in CSD_PONZEX [5], and [NdL^Me4a^(NO_3_)_2_]^+^ in CSD_SICCOX [8], with much lower values (about 2.1) for CShMs. The case of CSD_SICCOX has been commented on above, as it displays an unusual wave conformation (Figure 5), which significantly preserves the disposition of the *meso* form of the free L^Me4a^ ligand [8,12,18,21]. Regarding the neutral complex [LaL^cyh2^(NO_3_)_3_] in CSD_PONZEX [5], it contains the largest metal center among those employed, and it is the only undecacoordinate complex with a monodentate nitrate donor and two bidentate chelate ones as ancillary ligands. Therefore, their steric hindrance is not comparable to that of other complexes. If we focus our attention only on the L^cyh2^ ligand, with a considerable variety of complexes, the tendency is more evident (Figure 6b).

Apart from the acetate ligand of [DyL(OAc)_2_]^+^ and the presence of a µ_2_:η^1^,η^2^ bridging carbonate anion in [Eu_2_(L^cyh2^)_2_(CO_3_)(H_2_O)_3_]^4+^ in CSD_FOFBUA [6], only complexes with nitrate or chloride salts were prepared with this kind of ligand. A difference can be inferred, and when chloride was the only anion present in the crystal, the trivalent metal centers only achieved an octacoordinated environment. However, with potential bidentate chelating donors such as acetate, nitrate, and carbonate, the larger lanthanoids tended to give rise to at least nona- or mostly decacoordinate complexes. In contrast, those metal ions with lower radii, even in the presence of nitrate, achieved only a nona- or an octacoordination. The only exception corresponds to [YbL^cyh2^(NO_3_)_2_]^+^, with [Yb(NO_3_)_5_]^2−^ counterions in CSD_YIPJIR [9]. This complex was obtained in a dry medium, but it transformed in the typical octacoordinated [YbL^cyh2^(H_2_O)_2_]^2+^ complex (CSD_YIPJOX) when in contact with water, after suffering hydrolysis. Importantly, this transformation also involves a change in the chirality of the donor N atoms [9].

All the known octacoordinate complexes display a butterfly-like disposition. With this spatial arrangement, the best fitting geometry is a *D_3h_* trigonal prism, but no correlation was found between the CShM values and the ionic Ln^III^ radii. For them, the macrocyclic ligand *R*,*R*,*R*,*R-*L^cyh2^ display a pseudo-*C_2_* symmetry in CSD_FOFBOU [6], CSD_RIVQIY, CSD_RIVPOD, CSD_RIVPUJ, CSD_RIVQAQ, CSD_RIVQOE, CSD_RIVQEU [7], and CSD_YIPJOX [9] and which strongly reminds of that of the *R*,*R*,*R*,*R* diasteroisomer of the free ligand solved in CSD_RIVPIX [7], also with *C_2_* symmetry. For all these complexes, the chirality of the *N*_4_ donor set is *S*,*S*,*S*,*S*, the most stable diasteroisomer in the presence of water, as commented above. The only exception to this chirality is our complex [DyL^Me2^Cl_2_]^+^, whose *N*_4_ donor set is *S*,*R*,*S*,*R*.

This latter heterochiral configuration of the four N donor atoms is also observed for almost all of the saddle-like complexes (nona- and decacoordinate), in this case even with the independence of the chirality of other stereocenters present in L^x^, as occurring both for the homochiral *R*,*R*,*R*,*R* L^cyh2^ diasteroisomers [5,6,7,9] and the heterochiral *R*,*R*,*S*,*S-*L^cyh2^ diasteroismer in CSD_YIPJUD [7]. Again, the undecacoordinate [LaL^cyh2^(NO_3_)_3_] in CSD_PONZEX [5] is an exception, since the *S*,*S*,*S*,*S*-L^cyh2^ diasteroisomer displays an *R*,*R*;*R*,*S* configuration for the donor set.

### 2.4. Photophysical Properties of [DyL(OAc)_2_]OAc·7H_2_O·EtOH and [DyL^Me^_2_(Cl)_2_]Cl·2H_2_O

**Figure 7:**
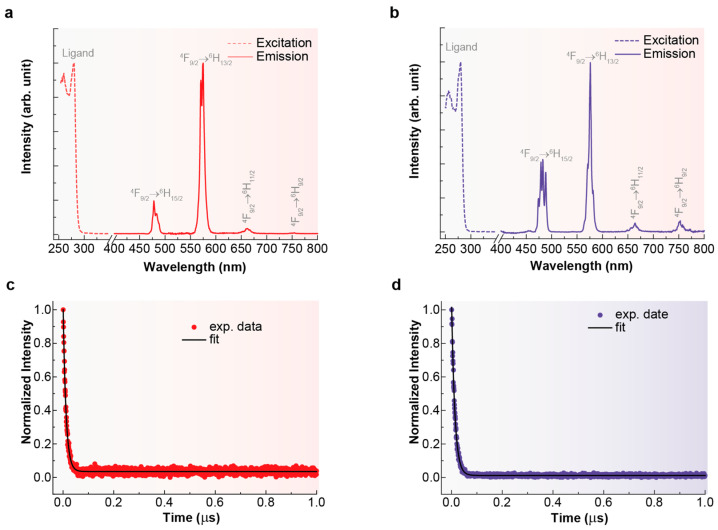
Excitation (dotted lines) and emission spectra (continuous lines) for [DyL(OAc)_2_]OAc·7H_2_O (**a**) and [DyLMe_2_(Cl)_2_]Cl·2H_2_O (**b**), recorded with *λ*_em_ = 576 nm and *λ*_ex_ = 280 nm, respectively. Decay curves recorded monitoring the emission at 576 nm (^4^F_9/2_ → ^6^H_13/2_) under 280 nm excitation (circles) and its fit to a single-exponential decay (black line) at room temperature for [DyL(OAc)_2_]OAc·7H_2_O (**c**) and [DyLMe_2_Cl_2_]Cl·2H_2_O (**d**).

The excitation spectra of the complexes [DyL(OAc)_2_]OAc·7H_2_O and [DyL^Me^_2_(Cl)_2_]Cl·2H_2_O were recorded while monitoring the ^4^F_9/2_ → ^6^H_13/2_ transition at 576 nm in 10^−3^ M methanolic solution. Both spectra are shown in Figure 7.

In both cases, the excitation spectra consist of a broad band with a maximum at 280 nm, which can be attributed to the π → π* transition centered on the aromatic units of the ligands [43]. This observation indicates that the emission is sensitized by the ligand. Emission spectra for both compounds in solution (Figure 7) were recorded using an excitation wavelength of 280 nm, consistent with the maxima observed in the excitation spectra. Consequently, the luminescence of [DyL(OAc)_2_]OAc·7H_2_O·EtOH and [DyL^Me2^(Cl)_2_]Cl·2H_2_O gives rise to a set of distinctive narrow bands characteristic of Dy^3+^, located between 400 and 800 nm. These bands correspond to the ^4^F_9/2_ → ^6^H*_J_* intra-*4f* transitions (*J* = 15/2, 13/2, 11/2, and 9/2) within the visible spectral region, the most intense being at 576 nm (*J* = 13/2), as expected. Comparison between the spectra of [DyL(OAc)_2_]OAc·7H_2_O·EtOH and [DyL^Me2^(Cl)_2_]Cl·2H_2_O reveals that differences in the symmetry of their coordination polyhedral lead to a redistribution of intensity in the split emission bands associated with the ^4^F_9/2_ → ^6^H_15/2, 13/2_ transitions. Additionally, the emission bands in the spectrum of [DyL^Me2^(Cl)_2_]Cl·2H_2_O are observed to be split, indicative of a less ordered crystalline structure compared to [DyL(OAc)_2_]OAc·7H_2_O·EtOH. Specifically, the band centered at 480 nm shows a stronger dependence on crystal symmetry compared to the bands at 576 nm, 664 nm, and 752 nm.

Luminescence lifetimes (τ) are depicted in Figure 7. Fitting the decay curve, monitored through the ^4^F_9/2_ → ^6^H_13/2_ transition, with a simple exponential function revealed a characteristic decay time of the order of μs for the Dy^3+^ emitting state (^4^F_9/2_). This value aligns closely with observations from other mononuclear dysprosium complexes [44]. As shown in Table 2, [DyL^Me2^(Cl)_2_]Cl·2H_2_O exhibits a slightly longer luminescence lifetime for visible emission compared to the corresponding values of [DyL(OAc)_2_]OAc·7H_2_O·EtOH, in agreement with its higher quantum yield. This difference can be attributed to variations in polyhedral symmetry [45]. In addition, the quantum yield of both compounds could seem quite low but remain quite high compared with those found for other dysprosium complexes [46]. This apparently relatively low yield can be explained by the much closer spacing of electronic energy levels in Dy^III^ compared to the highly luminescent Ln^III^ ions [47].

## 3. Materials and Methods

All chemical reagents were purchased from commercial sources and used as received, without further purification. Elemental analyses of C, H, and N were performed on a Flash Smart analyzer made by Thermo Fisher Scientific (Waltham, MA, USA). The ^1^H-NMR spectra of L^N6^ and L^N6Me^ were recorded on a Varian Inova 400 spectrometer (Agilent, Santa Clara, CA, USA).

### 3.1. Synthesis

The hexadentate amine ligands L and L^Me2^ were obtained as previously reported [18,19] and satisfactorily characterized by ^1^H-NMR spectroscopy and elemental analyses (L). Single crystals of the free hydrated ligand L·12H_2_O could be obtained by recrystallizing the crude product in water at a low temperature (~5 °C). When the crystals of L·12H_2_O were dried in an oven, they lost water to yield L.

L:^1^H-NMR (400 MHz, CDCl_3_, δ/ppm): 7.53 (t, 2H, H_aromatic_), 7.04 (d, 4H, H_aromatic_), 3.87 (s, 8H, H_CH2-py_), 2.82 (s, 8H, H_CH2-N_), 2.67 (s, 4H, H_NH_). Colorless single crystals were obtained by solving the white solid in a large amount of distilled water and leaving the solution in the fridge at ~5 °C for 14 h. M.W.: 326.43 g/mol. Anal. calcd. for C_18_H_26_N_6_ (%): C 66.23, N 25.75, H 8.03. Found: C 65.91, N 25.85, H 8.15.

L^Me2^: ^1^H-NMR (400 MHz, CDCl_3_, δ/ppm): L^N6Me^: ^1^H-NMR (400 MHz, CDCl_3_, δ/ppm): 7.53 (t, 2H, H_aromatic_), 7.16 (d, 2H, H_aromatic_), 7.03 (d, 2H, H_aromatic_), 3.73 (s, 4H, H_CH2-py_), 3.57 (s, 4H, H_CH2-py_), 2.73 (t, 4H, H_CH2-N_), 2.59 (t, 4H, H_CH2-N_), 2.27 (s, 6H, H_CH3_).

[DyL(OAc)_2_]OAc·7H_2_O·EtOH: To a solution of L^N6^ (0.100 g, 0.306 mmol) in chloroform (20 mL), a solution of dysprosium acetate tetrahydrate (0.126 g, 0.306 mmol) in methanol (10 mL) was added. The resultant yellow solution was stirred for 6 h, and the solvent was removed under vacuum, obtaining a green oil. Diethyl ether and dichloromethane were added, and the mixture was stirred for 4 h. Then, the solvents were removed under vacuum to dryness, obtaining a pale-yellow solid. The recrystallization of the pale-yellow solid in acetonitrile yielded single crystals of 1·7H_2_O. The crystals lost some of the water and ethanol solvate molecules on drying in an oven, yielding [DyL(OAc)_2_]OAc·2H_2_O. Yield: 0.085 g (40%). M.W.: 702.19 g/mol. Anal. calcd. for C_24_H_39_DyN_6_O_8_ (%): C 41.05, N 11.97, H 5.60. Found: C 40.91, N 12.05, H 5.54.

[DyL^Me2^Cl_2_]Cl·2H_2_O: to a solution of L^Me2^ (0.040 g, 0.113 mmol) in acetonitrile (20 mL), dysprosium chloride hexahydrate (0.043 g, 0.113 mmol) and methanol (10 mL) were added. The mixture was refluxed for 4 h, and then the solvent was removed under vacuum up to half of its initial volume. Colorless single crystals were isolated by layering the solution with diethyl ether in the fridge at ~5 °C. Yield: 0.030 g (40%). M.W.: 659.38 g/mol. Anal. calcd. for C_20_H_34_Cl_3_DyN_6_O_2_ (%): C 36.43, N 12.75, H 5.20. Found: C 36.51, N 12.85, H 5.34.

### 3.2. Crystallographic Refinement and Structure Solution

Crystal data and some details of the refinement are given in Table S1. Crystals suitable for the single X-ray diffraction of L·12H_2_O, [DyL(OAc)_2_]OAc·7H_2_O·EtOH and [DyL^Me2^(Cl)_2_]Cl·2H_2_O were obtained as previously described. Data were collected at 100 K on a Bruker D8 VENTURE PHOTON III-14 diffractometer, employing graphite monochromatized Mo-kα (λ = 0.71073 Å) radiation. Multi-scan absorption corrections were applied using SADABS [48]. These structures were solved using standard direct methods, employing SHELXT [49], and then refined by full-matrix least-square techniques on *F*^2^, using SHELXL from the program package SHELX-2018 [50].

All nonhydrogen atoms corresponding to the complexes were refined anisotropically, but in some cases, disordered atoms or solvates with low occupation sites were isotropically treated. Hydrogen atoms bonded to C atoms were typically included in the structure factor calculations in geometrically idealized positions. Non disordered hydrogen atoms attached to oxygen and/or nitrogen atoms, with partial occupation of 1, were mostly located in the corresponding Fourier maps, with the intention of revealing the hydrogen bonding scheme. In these cases, either they were freely refined or with thermal parameters derived from their parent atoms. When these H atoms could not be located in the Fourier map, they were fixed at reasonable positions.

### 3.3. Photophysical Properties

The spectrophotometric characterization was made by preparing stock solutions of [DyL(OAc)_2_]OAc·7H_2_O·EtOH and [DyL^Me2^(Cl)_2_]Cl·2H_2_O in methanol (ca. 10^−3^ M). All the measurements were taken at 298 K.

Photoluminescence spectroscopy: The excitation and emission spectra of the samples were recorded at room temperature (25 °C) using a FluoTime 300 spectrofluorometer (FT300, PicoQuant, Berlin, Germany ) equipped with a double additive emission monochromator featuring a diffraction grating with 1200 grooves mm^−1^, blazed at 500 nm (reciprocal linear density of 1.4 nm mm^−1^). A 300 W Xe lamp served as the excitation source. Emission spectra were corrected for the detection and optical spectral response of the spectrofluorometer, while excitation spectra were corrected for the spectral distribution of the lamp intensity using a photodiode reference detector. Both excitation and emission spectra were recorded with an integration time of 400 ms and a resolution of 0.5 nm. Emission decay profiles were also recorded using the same spectrofluorometer at room temperature, with a 10 W Xe flash lamp as the excitation source and a temporal resolution of 0.6 μs. The emission lifetimes (τ) were calculated using the Origin 2018 software (OriginLab, Northampton, MA, USA) by fitting a mono-exponential decay function (ExpDec1) to the normalized experimental decay curves of the Dy^3+^ compounds.

Emission Quantum Yield: The absolute emission quantum yields (q) were measured at room temperature (298 K) using a Quantaurus quantum yield measurement system (QY Plus C13534, by Hamamatsu, Shizuoka, Japan) with a 150 W Xenon lamp coupled to a monochromator for wavelength discrimination, an integrating sphere as sample chamber, and two multi-channel analyzers for signal detection in the visible spectral range. The values of q for the downshifting emission of Dy^3+^ correspond to the integration over the 400−800 nm spectral range under 280 nm excitation. The reported values present an accuracy of 10%, according to the manufacturer.

## 4. Conclusions

This paper describes the synthesis and crystallographic characterization of the first lanthanoid complex of the L ligand, the novel [DyL(OAc)_2_]OAc·7H_2_O·EtOH, and a rare example of a lanthanoid complex with the L^Me2^ donor, [DyL^Me2^(Cl)_2_]Cl·2H_2_O, thus increasing the very limited number of Ln^III^ complexes containing flexible hexaazatetramine macrocycles with acyclic spacers between the *N*_3_ moieties. Comparing both complexes with the only two previously reported lanthanoid coordination compounds of L^N6Me^, we conclude that ancillary ligands play a fundamental role in the coordination index of the metal ion, as well as in ligand folding. Thus, the presence of ancillary bidentate chelate donors favors a coordination index ≥ 9, whereas monodentate ancillary ligands are associated with a coordination index ≤ 9, despite their ostensibly smaller size. This trend is also observed in related complexes with cyclohexyl as a spacer.

Furthermore, the comparison of these compounds seems to indicate that low-bulk substituents on the nitrogen atoms, such as methyl groups, do not appear to induce significant changes in the folding of the *N*_6_-donor, and despite the limited number of complexes with this type of ligand and the variations in ancillary donors and ion sizes, we cannot state this categorically. Accordingly, further studies are needed in this field.

Other important conclusions are drawn from the analysis of related complexes. Chirality influences the predisposition of the ligands to adopt different spatial arrangements but is not a determining factor. In this sense, the ligands are especially adequate to allocate small 3*d* ions as Ni^II^; according to theoretical calculations, the smaller the ionic radii, the lower the deviation from an ideal octahedral disposition of the *N*_6_ donor set. Accordingly, lanthanoids appear too small for a totally planar and regular hexagonal disposition of L^x^. Thus, the higher the Ln^III^ radii are, the lower the deviation from the ideal. Finally, no correlation was found between the values of the Ln^III^ radii and the deviations from a *D_3h_* trigonal prism, which is the most accurate geometry found for the donor atoms of macrocycles in octacoordinate complexes characterized crystallographically.

The solution photophysical studies show that both compounds are luminescent and that the macrocyclic ligands L and L^Me2^ act as efficient sensitizers for energy transfer to the nearby Dy^3+^ ion.

## Figures and Tables

**Table 2:** Luminescence lifetimes (τ) monitoring the emission at 576 nm (^4^F_9/2_ → ^6^H_13/2_) under 280 nm excitation and absolute emission quantum yield (q) in the visible spectral range under excitation at 280 nm, measured at room temperature, for the Dy^3+^ complexes.

Compound	τ (μs)	R^2^	q (%)
[DyL(OAc)_2_]OAc·7H_2_O·EtOH	11.2 ± 0.1	0.98	1.70 ± 0.17
[DyL^Me2^(Cl)_2_]Cl·2H_2_O	12.9 ± 0.6	0.99	2.10 ± 0.21

## Data Availability

Data are contained within the article or Supplementary Materials. CCDC 2323338-2323340 contains the supplementary crystallographic data for this paper. These data can be obtained free of charge via www.ccdc.cam.ac.uk/data_request/cif, by emailing data_request@ccdc.cam.ac.uk, or by contacting The Cambridge Crystallographic Data Centre, 12 Union Road, Cambridge CB2 1EZ, UK; Fax: +44-1223-336033.

## Supplementary Materials

The following supporting information can be downloaded at: https://www.mdpi.com/article/10.3390/ijms25126802/s1.
