# Supplementary material for: Exploring the Spatial Arrangement of Simple 18-Membered Hexaazatetraamine Macrocyclic Ligands in Their Metal Complexes
Source: Int J Mol Sci. 2024 Jun 20;25(12):6802. doi: 10.3390/ijms25126802 (PMC11203486; doi:10.3390/ijms25126802)
Supplement: Supplementary file 1 [file ijms-25-06802-s001.zip › ijms-3041135-supplementary.pdf]

Supplementary Material

Exploring the Spatial Arrangement of Simple 18-Membered Hexaazatetraamine Macrocyclic Ligands in Their Metal Complexes

Julio Corredoira-Vázquez [1,2,3], Cristina González-Barreira [1], Jesús Sanmartín-Matalobos [1,3], Ana M. García-Deibe [1,*] and Matilde Fondo [1,*]

[1] Departamento de Química Inorgánica, Facultade de Química, Campus Vida, Universidade de Santiago de Compostela, 15782 Santiago de Compostela, Spain; julio.corredoira.vazquez@usc.es (J.C.-V.); cristina.gonzalez.barreira@rai.usc.es (C.G.-B.); jesus.sanmartin@usc.es (J.S.-M.)
[2] Phantom-g, CICECO—Aveiro Institute of Materials, Department of Physics, University of Aveiro, 3810-193 Aveiro, Portugal
[3] Institute of Materials (iMATUS), Universidade de Santiago de Compostela, 15782 Santiago de Compostela, Spain
* Correspondence: ana.garcia.deibe@usc.es (A.M.G.-D.); matilde.fondo@usc.es (M.F.)

Figure S1. Two examples of different conformations for the CPy-CH2-NH(2)-CH2 branches of acetylpyridne residues S2

Table S1. Crystal data and structure refinement for L·12H_2O, [DyL(OAc)$_2$]OAc·7H_2O·EtOH and [DyL^{Me2}(Cl)$_2$]Cl·2H_2O. S3

Table S2. Main bond distances (Å) and angles (º) for L·12H_2O. S4

Table S3. Some relevant features for lanthanoid complexes of the hexaaza tetraamine ligands crystallographically characterized. S5

Table S4. Some relevant features for 3*d* metal complexes of the hexaaza tetraamine ligands crystallographically characterized. S7

Table S5. Main bond distances (Å) and angles (°) for [DyL(OAc)$_2$]OAc·7H_2O·EtOH and [DyL^{Me2}(Cl)$_2$]Cl·2H_2O. S8

Table S6. SHAPE v2.1. Continuous Shape Measures Calculation (c) 2013 Electronic Structure Group, Universitat de Barcelona. S9

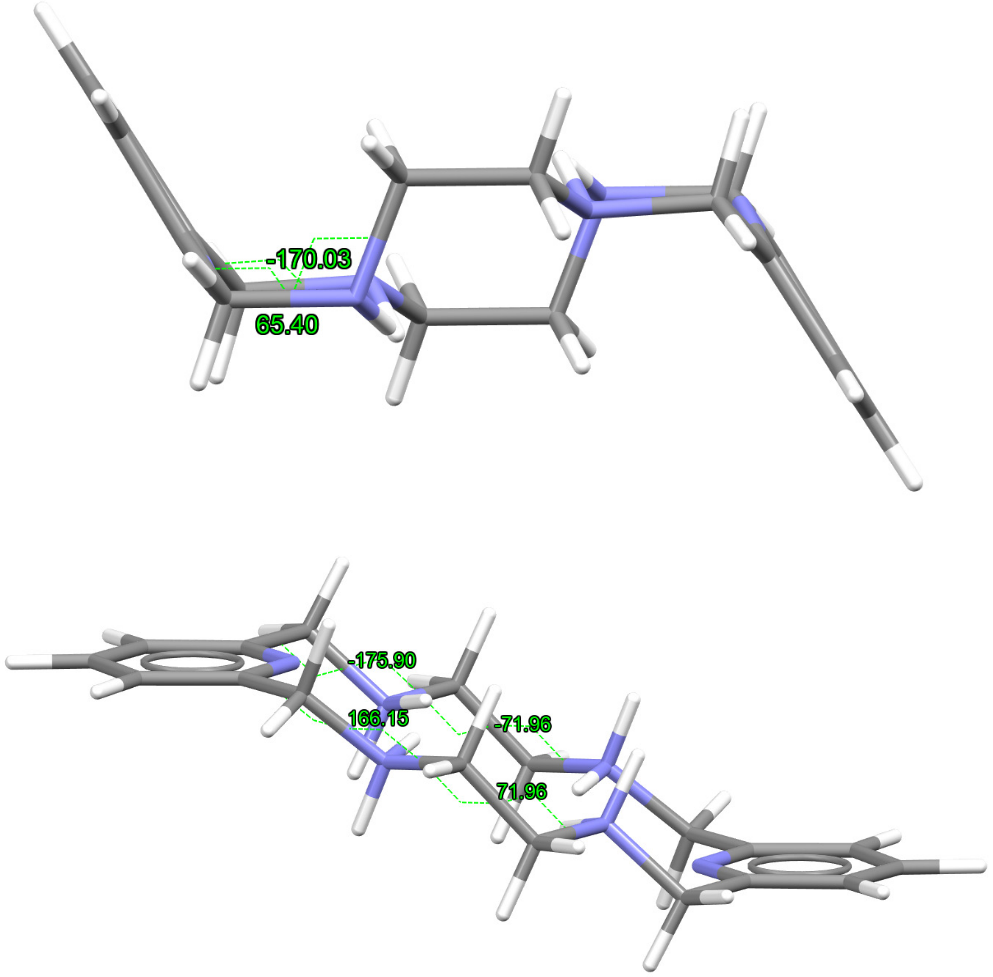

Figure S1. Two examples of different conformations for the C_{Py}-CH_2-$NH_{(2)}$-CH_2 branches of acetylpyridne residues: (Top): Gauche and alternate conformations found for L in L·$12H_2O$ (this work); and bottom: Alternate and alternate for ZUVKUW [21].

Table S1. Crystal data and structure refinement for L^{N6}·12H_2O, [DyL(OAc)$_2$]OAc·7H_2O·EtOH and [DyL^{Me2}(Cl)$_2$]Cl·2H_2O.

	L·12H_2O	**1**·7H_2O·EtOH	**2**·2H_2O
Empirical formula	$C_{18}H_{50}N_6O_{12}$	$C_{24}H_{49}DyN_6O_{13}$	$C_{20}H_{34}Cl_3DyN_6O_2$
Molecular weight	542.64	792.19	659.38
Crystal system	Triclinic	Triclinic	Monoclinic
Space group	*P-1*	*P-1*	$P2_1/n$
Wavelength (Å)	0.71073	0.71073	0.71073
Crystal size (mm^3)	0.25 x 0.15 x 0.14	0.10 x 0.05 x 0.02	0.13 x 0.08 x 0.02
Color, shape	Colorless, prism	Pale yellow, plate	Colorless, plate
T (K)	100	250	100
a (Å)	7.5502(4)	9.3422(4)	10.3146(8)
b (Å)	9.6015(5)	12.8831(7)	16.4037(13)
c (Å)	10.6416(5)	14.2999(7)	14.8724(11)
α (°)	75.945(2)	78.827(2)	90
β (°)	78.182(2)	80.662(2)	91.920(3)
γ (°)	85.489(2)	88.660(2)	90
Volume (Å^3)	732.15(6)	1666.03(14)	2515.0(3)
Z	1	2	4
Absorpt. coef. (mm^{-1})	0.102	2.310	3.319
Reflections collected	26696	98996	155377
Independent reflections	3631 [R_{int} = 0.0461]	8256 [R_{int}= 0.0842]	13578 [R_{int} = 0.0445]
Data / restraints / param.	3631 / 0 / 163	8256 / 0 / 400	7688 / 0 / 301
Final *R* indices [I>2$\sigma(I)$]	R_1 = 0.0420<br>wR_2 = 0.1069	R_1 = 0.0315<br>wR_2 = 0.0600	R_1 = 0.0159<br>wR_2 = 0.01594
R indices (all data)	R_1 = 0.0603<br>wR_2 = 0.1151	R_1 = 0.0424<br>wR_2 = 0.0636	R_1 = 0.0190<br>wR_2 = 0.0402

Table S2. Main bond distances (Å) and angles (°) for L·12H_2O.

C1-N1	1.4752(17)	C1-C9#1	1.5142(18)
C2-N1	1.4771(17)	C2-C3	1.5049(19)
C3-N2	1.3405(17)	C3-C4	1.3932(19)
C7-N2	1.3442(17)	C4-C5	1.385(2)
C8-N3	1.4765(17)	C5-C6	1.388(2)
C9-N3	1.4703(17)	C6-C7	1.3886(18)
		C7-C8	1.5117(19)
N1-C1-C9#1	110.94(11)	C4-C3-C2	121.82(12)
N1-C2-C3	109.65(11)	C5-C4-C3	118.52(13)
N2-C3-C4	122.24(12)	C4-C5-C6	119.32(12)
N2-C3-C2	115.84(11)	C5-C6-C7	118.83(13)
N2-C7-C6	121.99(12)	C6-C7-C8	122.91(12)
N2-C7-C8	115.01(11)	C1-N1-C2	110.97(10)
N3-C8-C7	112.56(10)	C3-N2-C7	119.02(11)
N3-C9-C1#1	111.61(11)	C9-N3-C8	112.02(10)

#1: -x+1,-y+2,-z

Table S3. Some relevant features for lanthanoid complexes of the hexaaza tetraamine ligands crystallographically characterized.

Complex / Counterion / Coord. No.	Chirality	Conformation of the ligand	Ln^{III} Radius /Å [31-33]	Deviation from HP-6 ChMS / best ChMS geom.*	CSD-REFCOD Ref.
$[YL^{cyh2}(H_2O)Cl]^{2+}$/<br>Cl^-<br>8	C: *R,R,R,R*,<br>N: *S,S,S,S*	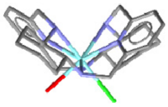<br>Butterfly	1.159 [33]	20.564<br>10.433 TPR	RIVQIY<br>[7]
$[LaL^{cyh2}(NO_3)_3]$<br><br>11	C: *S,S,S,S*<br>N: *R,R; R,S**	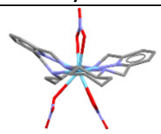<br>Saddle	1.16 [31],<br>1,250 [32]	2.123	PONZEX<br>[5]
$[CeL^{Me2}(NO_3)_2]^+$/<br>NO_3^-<br>10	N:R',S; S,R'<br>'Me<br>substituted	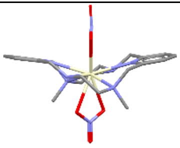<br>Saddle	1.143 [31],<br>1.220 [32]	4.552	DIJZEE<br>[4]
$[PrL^{cyh2}H_2O(NO_3)]^{2+}$ /<br>NO_3^-<br>10	C: *R,R,R,R*,<br>N: *R,S;S,R*	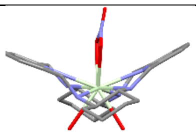<br>Saddle	1.126 [31],<br>1.200 [32]	Pr1: 4.863<br>Pr2: 4.905	YIPHUB<br>[9]
$[NdL^{Me4a}(NO_3)_2]^+$ /<br>NO_3^-<br>10	C: *R,R;S,S*,<br>N: *S,S;R,R*,	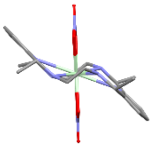<br>Wave	1.109 [31],<br>1.175 [32]	2.093	SICCOX<br>[8]
$[SmL^{Me2}(NO_3)_2]^+$/<br>NO_3^-<br>10	N: *R',S; S,R'*<br>'substituted	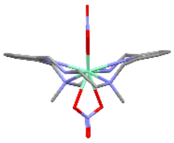<br>Saddle	1.079 [31],<br>1.140 [32]	4.967	DIJZII<br>[4]
$[SmL^{cyh2}(H_2O)_2(NO_3)]^{2+}$/<br>$NO_3^-$<br>10	C: *R,R,R,R*,<br>N: *R,S;S,R*	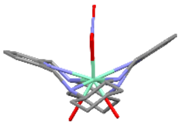<br>Saddle		Sm1: 5.164 Sm2:<br>5.137	RIVQUK<br>[7]
$[Eu_2(L^{cyh2})_2(H_2O)_3(CO_3)]^{4+}$<br>/ Cl^-<br>9	C: *R,R,R,R*,<br>N: *R,S;S,R*	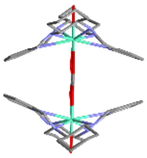<br>Saddle	1.066 [31],<br>1.120 [32]	5.043	FOFBUA<br>[6]
$[EuL^{cyh2}(H_2O)(NO_3)]^{2+}$ /<br>NO_3^-<br>10	C: *R,R,R,R*,<br>N: *R,S;S,R*	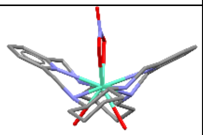<br>Saddle		Eu1: 5.197<br>Eu2: 5.238	YIPJAJ<br>[9]

$[EuL^{cyh2}(H_2O)(NO_3)]^{2+}$ / NO_3^- 9	C: *R,R; S,S* N: *R,S;S,R*	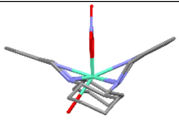 Saddle		5.151	YIPJUD [7]
$[TbL^{cyh2}(H_2O)(NO_3)]^{2+}$/ NO_3^- 9	C: *R,R,R,R,* N: *R,S;S,R*	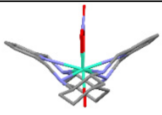 Saddle	1.040 [31], 1.090 [32]	5.329	RIVRAR [7]
$[DyL(OAc)_2]^+$ / AcO^- 10	N: *R,S;S,R*	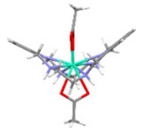 Saddle	1.027 [31], 1.075 [32]	5.559	This work
$[DyL^{cyh2}(NO_3)_2]^+$ / $[Dy(NO_3)_5]^{2-}$ 10	N: *R,S;R,S* N: *R,S;S,R*	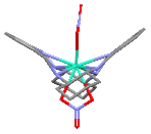 Saddle		Dy1: 6.086 Dy2: 5.781	YIPJEN [9]
$[DyL^{cyh2}(H_2O)Cl]^{2+}$/ Cl^- 8	C: *R,R,R,R,* N: *S,S,S,S*	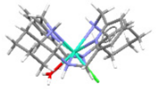 Butterfly		20.479 10.542/TPR	FOFBOU [6]
$[DyL^{Me2}(Cl)_2]^+$ / Cl^- 8	N: *R',S; S,R'* 'substituted	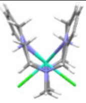 Butterfly		20.430 / 9.546 TPR	This work
$[HoL^{cyh2}(H_2O)(NO_3)]^{2+}$ / NO_3^- 9	C: *R,R,R,R,* N: *S,S,S,S*	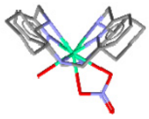 Butterfly	1.015 [31], 1.055 [32]	11.871/TPR	RIVPOD [7]
$[ErL^{cyh2}(H_2O)(NO_3)]^{2+}$ / NO_3^- 9	C: *R,R,R,R,* N: *S,S,S,S*	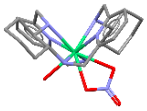 Butterfly	1.004 [31], 1.040 [32]	11.875/TPR	RIVPUJ [7]
$[TmL^{cyh2}(H_2O)_2]^{3+}$ / NO_3^- 8	C: *R,R,R,R,* N: *S,S,S,S*	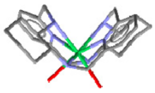 Butterfly	0.994 [31], 1.025 [32]	21.873 11.375/TPR	RIVQAQ [7]
$[YbL^{cyh2}(NO_3)_2]^+$ / $[Yb(NO_3)_5]^{2-}$ 10	C: *R,R,R,R,* N: *R,S;S,R*	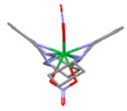 Saddle	0.985 [31], 1.010 [32]	Yb1: 6.317 Yb2: 6.036	YIPJIR [9]
$[YbL^{cyh2}(H_2O)Cl]^{2+}$/ Cl^- 8	C: *R,R,R,R,* N: *S,S,S,S*	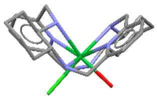 Butterfly		20.354 12.801/TPR	RIVQOE [7]
$[YbL^{cyh2}(H_2O)_2]^{2+}$ / NO_3^- 8	C: *R,R,R,R,* N: *S,S,S,S*	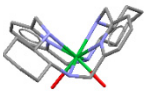 Butterfly		21.767 11.305/TPR	YIPJOX [9]
$[LuL^{cyh2}(H_2O)_2]^{2+}$ / NO_3^- 8	C: *R,R,R,R,* N: *S,S,S,S*	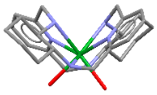 Butterfly	0.977 [31], 0.995 [32]	21.963 11.242/TPR	RIVQEU [7]

* Spacers separated by ";" if necessary ; HP-6 (1 D_{6h} Hexagon); TPR-6 (4 D_{3h} Trigonal prism)

Table S4. Some relevant features for *3d* metal complexes of the hexaazatetraamine ligands crystallographically characterized.

**Complex/ anion**		**Chirality**	**Conformation of the ligand**	**Deviation from HP6 ChMS / best ChMS geom.***	**CSD-REFCOD/ Ref**
$[MnL]^{2+}$/ Br_4Mn^{2-}	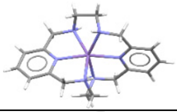	N: *R,R,R,R*; Mn: *P*	twisted	25.391 6.925/OC	IGESIX [11]
$[CoL^{Me4}]^{2+}$/ Cl_4Co^{2-}	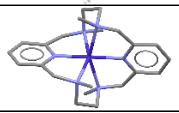	N: *R,R,R,R*; Co: *P*	twisted	29.746 4.353/OC	BIHCEA10 [10]
$[NiL^{Me4a}]^{2+}$ / ClO_4^-	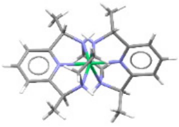	C; *R,S; R,S*;** N: *R,R,R,R*, Ni: *P*	twisted	30.474 3.925/OC	KUBCUG [12]
$[NiL]^{2+}$ / ClO_4^-	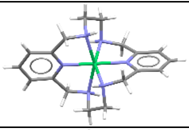	N: *S,S,S,S* ; Ni: *M*	twisted	30.218 3.803/OC	WUCYEY [15]
$[CuL]^{2+}$/ ClO_4^-	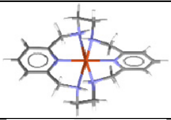	N: *S,S,S,S* ; Cu: *M*	twisted	29.144 4.578/OC	WUCYIC [15]
$[CuL^{Me4}]^{2+}$/ Cl_4Cu^{2-}	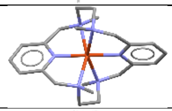	N: *R,R,R,R* Cu: *P*	twisted	30.723 4.193/OC	BIHCIE10 [10]
$[ZnL]^{2+}$/ $CF_3O_3S^-$	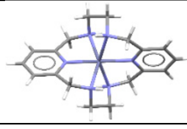	N *R,R,R,R*; Zn: *P*	twisted	27.611 5.266/OC	WIFWOX [13]
$[ZnL^{cyh2}]^{2+}$/ ClO_4^-	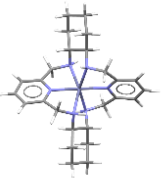	C: *R,R,R,R*; N: *S,S,S,S*; Zn: *P*	twisted	27.819 4.895/OC	PONYUM [5]
$[CdL^{cyh2}]^{2+}$/ ClO_4^-	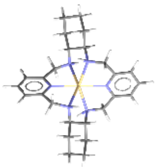	C: *S,S,S,S;* N: *R,R,R,R*, Cd: *M*	twisted	21.397 8.526/OC	PONZAT [5]
$[CdL]^{2+}$/ $CF_3O_3S^-$	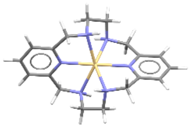	N: *S,S,S,S* Cd: *M*	twisted	21.503 8.744/OC	ZAXMUG [14]

* HP-6 (1 D_{6h} Hexagon); OC (Octahedron);
** Spacers separated by ";" if necessary.

Table S5. Main bond distances (Å) and angles (°) for $[DyL(OAc)_2]OAc{\cdot}7H_2O{\cdot}EtOH$ and $[DyL^{Me2}(Cl)_2]Cl{\cdot}2H_2O$.

	$[DyL(OAc)_2]OAc{\cdot}7H_2O{\cdot}EtOH$	$[DyL^{Me2}(Cl)_2]Cl{\cdot}2H_2O$
Dy1-N1	2.609(3)	2.6186(13)
Dy1-N2	2.536(2)	2.5079(13)
Dy1-N3	2.564(2)	2.5064(13)
Dy1-N4	2.644(3)	2.6326(13)
Dy1-N5	2.565(3)	2.4903(13)
Dy1-N6	2.559(3)	2.5096(13)
Dy1-O1	2.437(2)	-
Dy1-O2	2.504(2)	-
Dy1-O3	2.438(2)	-
Dy1-O4	2.577(2)	-
Dy1-Cl1	-	2.6523(4)
Dy1-Cl2	-	2.6196(4)
N1-Dy1-N4	177.29(8)	155.43(4)
N2-Dy1-N5	145.77(8)	104.82(4)
N3-Dy1-N6	148.28(8)	108.12(4)
Cl1-Dy1-Cl2	-	101.626(14)

Table S6. SHAPE v2.1. Continuous Shape Measures Calculation (c) 2013 Electronic Structure Group, Universitat de Barcelona.

Geometries coordination number 10

HD-10	13 D4h	Hexadecahedron (2:6:2) or (1:4:4:1)
TD-10	12 C2v	Tetradecahedron (2:6:2)
SDD-10	11 D2	Staggered Dodecahedron (2:6:2)
JSPC-10	10 C2v	Sphenocorona J87
JATDI-10	9 C3v	Augmented tridiminished icosahedron J64
JMBIC-10	8 C2v	Metabidiminished icosahedron J62
JBCSAPR-10	7 D4d	Bicapped square antiprism J17
JBCCU-10	6 D4h	Bicapped cube J15
PAPR-10	5 D5d	Pentagonal antiprism
PPR-10	4 D5h	Pentagonal prism
OBPY-10	3 D8h	Octagonal bipyramid
EPY-10	2 C9v	Enneagonal pyramid
DP-10	1 D10h	Decagon

Geometries coordination number 8

ETBPY-8	13 D3h	Elongated trigonal bipyramid
TT-8	12 Td	Triakis tetrahedron
JSD-8	11 D2d	Snub diphenoid J84
BTPR-8	10 C2v	Biaugmented trigonal prism
JBTPR-8	9 C2v	Biaugmented trigonal prism J50
JETBPY-8	8 D3h	Johnson elongated triangular bipyramid J14
JGBF-8	7 D2d	Johnson gyrobifastigium J26
TDD-8	6 D2d	Triangular dodecahedron
SAPR-8	5 D4d	Square antiprism
CU-8	4 Oh	Cube
HBPY-8	3 D6h	Hexagonal bipyramid
HPY-8	2 C7v	Heptagonal pyramid
OP-8	1 D8h	Octagon

1·7H_2O·EtOH

Structure [ML10]	HD-10	TD-10	SDD-10	**JSPC-10**	JATDI-10	JMBIC-100
	7.045,	4.325,	5.020,	**3.565**,	19.955,	7.562,
**JBCSAPR-10**	JBCCU-10	PAPR-10	PPR-10	OBPY-10	EPY-10	DP-1
**3.401**,	9.794,	12.575,	12.420,	13.551,	25.275,	36.968

2·2H_2O

Structure [ML8]	ETBPY-8	TT-8	JSD-8	**BTPR-8**	JBTPR-8	JETBPY-8
,	25.468,	13.941,	4.814,	**3.062**,	3.441,	27.792,
JGBF-8	**TDD-8**	SAPR-8	CU-8	HBPY-8	HPY-8	OP-8
14.761,	**2.479**,	3.486,	13.327,	17.158,	23.890,	29.436
